# Co-Infection with Marek’s Disease Virus and Reticuloendotheliosis Virus Increases Illness Severity and Reduces Marek’s Disease Vaccine Efficacy

**DOI:** 10.3390/v9060158

**Published:** 2017-06-21

**Authors:** Guo-Rong Sun, Yan-Ping Zhang, Lin-Yi Zhou, Hong-Chao Lv, Feng Zhang, Kai Li, Yu-Long Gao, Xiao-Le Qi, Hong-Yu Cui, Yong-Qiang Wang, Li Gao, Qing Pan, Xiao-Mei Wang, Chang-Jun Liu

**Affiliations:** Division of Avian Immunosuppressive Diseases, State Key Laboratory of Veterinary Biotechnology, Harbin Veterinary Research Institute, Chinese Academy of Agricultural Sciences, Harbin 150069, China; sgrshenhua@hotmail.com (G.-R.S.); zhyp_77@hvri.ac.cn (Y.-P.Z.); zlyi123321@126.com (L.-Y.Z.); lv6739533@163.com (H.-C.L.); 18021339037@163.com (F.Z.); likaihvri@163.com (K.L.); ylg@hvri.ac.cn (Y.-L.G.); qxl@hvri.ac.cn (X.-L.Q.); cuihy@hvri.ac.cn (H.-Y.C.); yqw@hvri.ac.cn (Y.-Q.W.); gaoli0820@163.com (L.G.); panqing20050101@126.com (Q.P.)

**Keywords:** Marek’s disease virus, reticuloendotheliosis virus, co-infection, pathogenicity, vaccine efficacy

## Abstract

Marek’s disease virus (MDV) and reticuloendotheliosis virus (REV) cause Marek’s disease (MD) and reticuloendotheliosis (RE), respectively. Co-infection with MDV and REV is common in chickens, causing serious losses to the poultry industry. However, experimental studies of such co-infection are lacking. In this study, Chinese field strains of MDV (ZW/15) and REV (JLR1501) were used as challenge viruses to evaluate the pathogenicity of co-infection and the influence of MD vaccination in chickens. Compared to the MDV-challenged group, the mortality and tumor rates increased significantly by 20.0% (76.7 to 96.7%) and 26.7% (53.3 to 80.0%), in the co-challenged group, respectively. The protective index of the MD vaccines CVI988 and 814 decreased by 33.3 (80.0 to 47.7) and 13.3 (90.0 to 76.7), respectively. These results indicated that MDV and REV co-infection significantly increased disease severity and reduced the vaccine efficacy. The MDV genome load showed no difference in the feather pulps and spleen, and pathogenicity-related MDV gene expression (*meq*, *pp38*, *vIL-8*, and *ICP4*) in the spleen significantly increased at some time points in the co-challenged group. Clearly, synergistic pathogenicity occurred between MDV and REV, and the protective efficacy of existing MD vaccines was attenuated by co-infection with Chinese field MDV and REV strains.

## 1. Introduction

Marek’s disease virus (MDV) is an oncogenic alphaherpesvirus that causes Marek’s disease (MD), a major viral oncosis that affects poultry health worldwide [1]. Infection of chickens with virulent strains of MDV causes a particularly serious group of severe symptoms, including T-cell lymphomas and solid visceral tumors, strong immunosuppression, and neurological disorders, leading directly to death or health complications in infected chickens [2,3]. The ability of MDV to replicate in the host is related to its pathogenicity, and the MDV genome load in infected chickens contributes to our understanding of the pathogenesis of MDV infection [4,5]. In addition, several MDV-encoded genes, including *meq* (MDV EcoRI-Q-encoded protein) [6,7,8], *pp38* (MDV phosphoprotein 38) [9,10], *vIL-8* (MDV-encoded CXC chemokine viral interleukin 8) [11,12], and *ICP4* (MDV infected-cell peptide 4) [13,14,15], play important roles in MDV pathogenesis. Vaccination with MD vaccines is the primary approach used to protect chickens against MD. Although the immune-protection mechanisms induced by MD vaccines are not fully understood currently, it is recognized that effective immunity to MD requires the involvement and coordinated activation of innate and adaptive immune responses [16]. In addition, it is postulated that vaccine-induced adaptive immunity plays a major role in providing protection against MD. However, due to the lymphotropic and highly cell-associated nature of MDV, T cell-mediated immune responses are thought to play a more important role than antibody-mediated responses [16,17]. Dramatic success has been achieved through the extensive use of MD vaccines, greatly reducing economic losses in the poultry industry [18,19]. Nevertheless, MD outbreaks have continued around the world in recent years, which is likely due to MDV evolution and co-infection with other viruses [20,21,22,23,24,25]. At present, the attenuated MDV strains CVI988 and 814 are considered the best vaccines against MD [26,27,28], and commercial CVI988 and 814 vaccines are widely used in China.

Reticuloendotheliosis virus (REV) is an oncogenic and immunosuppressive retrovirus that causes reticuloendotheliosis (RE) [29], an avian disease mainly characterized by immunosuppression, runting–stunting syndrome, and chronic lymphomas [30,31]. REV has extensive avian hosts, including chickens, turkeys, ducks, mallards, geese, peafowl, pheasants, pigeons, Hungarian partridges, Chinese partridges, Attwater’s prairie chickens, and many other wild birds [32,33,34,35,36,37,38,39]. REV infection of susceptible hosts, such as chicks, usually causes atrophy of the thymus and bursa of Fabricius, impairing the development and immune system functions of infected hosts, resulting in the suppression of host immune responses to some avian vaccines [40,41,42]. In addition, REV can be present as a contaminant in a variety of poultry biologics and vaccines [43,44,45,46,47,48,49]. The wide range of host species and potential for contaminations with REV contribute to viral transmission. Previous serological surveys have revealed that the positive rate of REV was approximately 2.3–23.5% among commercial chicken and turkey flocks in the United States [50]. Likewise, REV infection is highly prevalent in China; the seropositive rate has even reached 30–40% in some regions, causing serious economic losses [51,52,53]. No REV vaccines are commercially available to control REV infection at present; thus, the control of REV mainly depends on the elimination of diseased chickens.

In addition to the losses caused by MDV or REV infection alone, several reports have described the co-infection of MDV and REV in Chinese chicken flocks [51,54,55,56,57]. According to epidemiological results obtained in our laboratory, the REV-positive rates in MDV-positive clinical samples ranged from 11.7 to 16.4% every year between 2010 and 2016, which suggests that co-infection with MDV and REV in Chinese chicken flocks is common and may be an important threat against poultry health. Previous findings have demonstrated that the humoral immune responses to an early MD vaccine strain (herpesvirus of turkeys, HVT) were drastically reduced by REV contamination [40]. In addition, previous researchers found that the REV-contaminated HVT vaccine conferred less protection against an MDV reference strain JM/102W [41]. However, the influences of co-infection with Chinese field strains of MDV and REV on disease progression and the efficacy of existing MD vaccines widely used today are poorly understood, experimentally. In this study, Chinese field strains of MDV (ZW/15) and REV (JLR1501) were used to study the pathogenicity of co-infection, and the protective effectiveness of two commercial MD vaccines (CVI988 and 814) widely used in China were evaluated to determine the influence on MD vaccination of co-infection in specific pathogen-free (SPF) chickens.

## 2. Materials and Methods 

### 2.1. Viruses and MD Vaccines

MDV strain ZW/15 and REV strain JLR1501 were separately isolated from two diseased Chinese chicken flocks in 2015 and identified by performing indirect immunofluorescence assays (IFAs) in chicken embryo fibroblasts (CEFs) with an MDV gE-specific monoclonal antibody [58] or a REV gp90-specific monoclonal antibody [59], respectively. The MD vaccines CVI988 and 814, which are widely used in China, were purchased from commercial suppliers.

### 2.2. PCR-Based Detection of MDV and REV

To further characterize the virus strains and MD vaccines used in this study and determine their purity, PCR amplification to detect MDV and REV was performed using reaction mixtures containing 10 µL Premix Taq^TM^ (TaKaRa Biotechnology Co., Ltd., Dalian, China), 1 µL forward and reverse primer (10 µM), 1 µL total DNA, and 7 µL sterile water in a final volume of 20 µL, with the following thermocycling profile: 1 cycle of 95 °C for 5 min, and 35 cycles of 95 °C for 30 s, 54 °C for 30 s, and 72 °C for 60 s, followed by cooling at 4 °C. Total DNA from infected CEFs was extracted using the AxyPrep BodyFluid Viral DNA/RNA Miniprep Kit (Corning Life Sciences Co., Ltd., Suzhou, China) following the manufacturer’s instructions, and used as a PCR template. The DNA of CEFs infected with MDV strain GA [60] and REV strain HLJR0901 [37] were used as positive controls for detecting MDV and REV, respectively, and DNA from uninfected CEFs was used as a mock control. This PCR method was also used to detect MDV and REV infection in chickens using DNA from their tissue samples collected from the animal experiments. The sequences of the primers used in these amplifications are shown in Table S1.

### 2.3. Design of Animal Experiments

One-day-old, SPF white Leghorn chickens were obtained from the Experimental Animal Center of the Harbin Veterinary Research Institute (HVRI) of the Chinese Academy of Agricultural Sciences. The birds were randomly numbered and divided into eight groups, then individually housed in negative pressure-filtered air isolators, and given free access to feed. On day one, the chickens in four groups were vaccinated with either CVI988 (two groups; *n* = 30 each) or 814 (two groups; *n* = 30 each) at a dose of 2000 plaque-forming units (PFUs) in 200 µL dedicated diluent provided by the manufacturers. In parallel, the other four groups were inoculated with 200 µL diluent. On day seven post-vaccination, one of the CVI988-vaccinated groups was challenged with 1000 PFUs of MDV ZW/15 in 200-µL diluent, while the second group vaccinated with CVI988 was inoculated with 1000 PFUs MDV ZW/15 and 10^4^ TCID_50_ (50% tissue culture infective dose) of REV JLR1501 in 200 µL diluent; at the same time, the same procedures were used for chickens in both 814-vaccinated groups. The four unvaccinated groups were treated as follows: (1) 1000 PFUs of MDV ZW/15 and 10^4^ TCID_50_ of REV JLR1501 in 200 µL diluent (co-challenged group; *n* = 55); (2) 1000 PFUs of MDV ZW/15 in 200 µL diluent (MDV-challenged control group; *n* = 55); (3) 10^4^ TCID_50_ of REV JLR1501 (REV-challenged control group; *n* = 15); (4) 200 µL diluent (negative control group; *n* = 15). All vaccinations were administered subcutaneously, and all viral challenges were administered intra-abdominally.

The birds were observed daily for signs of illness, and all birds that died during the experiment or were sacrificed humanely at the end-point of the experiment at 90 days post-challenge (dpc) were examined post-mortem, with appropriate samples obtained for diagnosis and histopathology. The MD status of the experimental animals was estimated as previously described [21]. In brief, all dead or sacrificed chickens were treated as MD-positive cases if they showed severe bursal and thymic atrophy; and/or rapid onset of T cell lymphomas that infiltrated lymphoid tissues, visceral organs, and peripheral nerves with severe clinical symptoms; and/or early-mortality syndrome without an MD lesion, but with an MDV-positive result by PCR-based detection (as described above) and by agar gel-precipitation testing using diagnostic MD reagents (WEIKE Biotechnology, Harbin, China). PCR detection of REV using DNA from chicken tissues was performed as described above, and enzyme-linked immunosorbent assays (ELISAs) were performed using the IDEXX REV Ab Test Kit (IDEXX Laboratories, Inc., Beijing, China) following the manufacturer’s instructions to determine the REV-infection status. Feather pulps were randomly plucked from five chickens in each group at 4, 7, 14, 21, 28, 35, and 42 dpc to determine the MDV genome load. Five chickens selected randomly in the co-challenge group and MDV-challenged control group were euthanatized humanely at 4, 7, 14, 21, and 28 dpc, and spleen samples were collected to determine the MDV genome load and study MDV gene expression.

The animal experiments were performed in a blinded manner. All animal experiments were evaluated and approved by the Animal Ethics Committee Review Board of the HVRI and performed in accordance with approved animal care guidelines and protocols (Approval No. SYXK [hei] 2011022).

### 2.4. Histopathological Examination

The tissues obtained at necropsy were fixed in 10% formaldehyde, routinely processed, and embedded in paraffin wax. The samples were cut approximately and then examined after hematoxylin and eosin staining. Then, the histopathological lesions were analyzed based on the optical density and area percent using Image Pro Plus 6.0 software (Media Cybernetics Inc., Rockville, MD, USA) with the “measure stain” function.

### 2.5. Real-Time Quantitative PCR and Reverse Transcription qPCR

The collected feather pulps and spleen tissues were homogenized in phosphate-buffered saline (PBS), and DNA was extracted as described above. The MDV *meq* gene was used as an MDV genome target gene and the chicken ovotransferrin gene was used as reference gene for the host cell. Real- time quantitative PCR (qPCR) detection was performed as previously described [61].

RNA from the collected spleen samples was extracted using RNAiso Plus (TaKaRa Biotechnology Co.) following the manufacturer’s instructions, and then cDNA was prepared from the RNA using ReverTra Ace qPCR RT Master Mix (TOYOBO Biotech Co., Ltd. Shanghai, China) and used as a template for reverse transcription qPCR (RT-qPCR). RT-qPCR was performed using the Premix Ex Taq^TM^ (Probe qPCR) Kit (TaKaRa Biotechnology Co.). The RT-qPCR mixture consisting of 12.5 µL Premix Ex Taq, 0.5 µL PCR forward and reverse primer (10 µM), 1 µL TaqMan Probe, and 1µL total cDNA, which was brought to a volume of 25 µL with 9.5 µL of added sterile water. The detection of specific targets was performed with following cycle profile: one cycle of 95 °C for 30 s, 40 cycles of 95 °C for 5 s and 60 °C for 35 s (detecting fluorescent signal), and one cycle of 50 °C for 30 s. Using this RT-qPCR method, the expression levels of four MDV pathogenicity-related genes (*meq*, *pp38*, *vIL-8*, and *ICP4*) in the spleen tissues of the MDV-challenged control chickens and MDV and REV co-challenged chickens were detected. The sequences of the primers and probes used to amplify and detect the MDV genes and reference host gene (*28S*) are shown in Table S2.

### 2.6. Statistical Analysis

The vaccine protective index (PI) was calculated as previously described [62], using the following equation: PI = ([%MD in unvaccinated chickens − %MD in vaccinated chickens]/[%MD in unvaccinated chickens]) × 100. The absolute numbers of the MDV genome per million cells from the collected feather pulps and spleen tissues were calculated as previously described [61] and were normalized using the following formula: normalized MDV viral load = log_10_ ([MDV genome copy number/chicken genome copy number] × 10^6^). The MDV gene-expression results were normalized to expression of the *28S* gene, and differences in the expression of MDV genes between the MDV and REV co-challenged group and the MDV-challenged control group are expressed as fold-changes on a logarithmic scale (base: 10). Viral load, MDV gene expression, and survival data were analyzed using GraphPad Prism (Version 7.03; GraphPad Software, Inc., La Jolla, CA, USA). Comparisons of the viral-load and MDV gene-expression data between two groups for each time point were performed using multiple *t* tests (Holm–Sidak method, alpha = 0.05; GraphPad Prism). Survival curves between two groups were compared using a log-rank test (Mantel–Cox) with GraphPad Prism. Differences were considered statistically significant at *p* < 0.05.

## 3. Results

### 3.1. MDV Strain ZW/15 and REV Strain JLR1501

Cytopathogenic effects (CPEs) in MDV ZW/15-infected CEFs were observed and confirmed by IFA using a MDV gE-specific monoclonal antibody (Figure S1A), and REV JLR1501 infection was determined by IFA using a REV gp90-specific monoclonal antibody (Figure S1B).

### 3.2. PCR Identification and Purity of the Viruses and MD Vaccines

When detecting MDV, the PCR product of ZW/15 *meq* gene was 786 base pairs (bp) long, which was consistent with that of the reference MDV strain GA, while those of CVI988 and 814 were 963 bp long because they have a 177 bp insertion in the *meq* gene (Figure S2A). When detecting REV, the PCR product (383 bp) of JLR1501 was consistent with that of reference REV strain HLJR0901 (Figure S2B). Furthermore, the MDV strain ZW/15 and vaccine strains CVI988 and 814 were free of REV, and the REV strain JLR1501 was free of MDV, based on the PCR results.

### 3.3. Animal Experiments of REV

PCR and ELISA results showed that all negative control and MDV-challenged groups (unvaccinated or vaccinated) were REV-free, and the positive rates of REV infection in the REV-challenged, and the MDV and REV co-challenged (unvaccinated or vaccinated) groups were similar (90.0, 93.3, and 90.0%, respectively). These results suggested that the status of MDV infection or MD vaccination had no effect on the REV infection rate. In addition, the infected chickens in the REV-challenged group mainly developed non-neoplastic syndromes with runting–stunting and feather dysplasia during the experimental period.

### 3.4. MD Incidence and Mortality

During the animal experiments, no chickens in the negative control group or REV-challenged group were MD positive, while all chickens in the MDV-challenged, and the MDV and REV co-challenged unvaccinated groups, developed MD. Furthermore, the mortality of MDV-challenged unvaccinated chickens was 76.7%, while that of the MDV and REV co-challenged unvaccinated group was significantly higher (96.7%). No chickens died in the negative control group or REV-challenged group. These results suggested that the severity of disease to SPF chickens caused by co-infection with MDV and REV was more serious than with MDV or REV infection alone. These results are summarized in Table 1.

**Figure 1:**
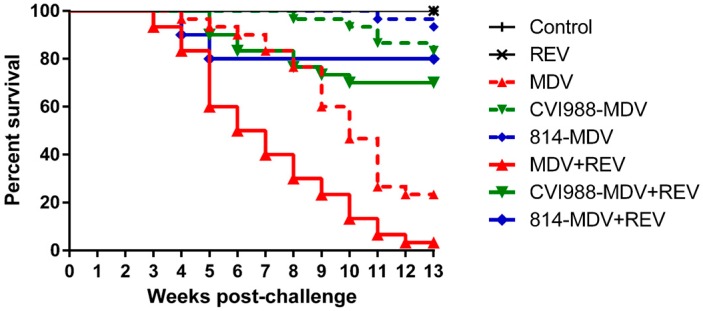
Survival curves for each experimental group. Comparison of survival curves between the (i) MDV-challenged control group and co-challenged group, (ii) CVI988-vaccinated MDV-challenged group and CVI988-vaccinated co-challenged group, and (iii) 814-vaccinated MDV-challenged group and 814-vaccinated co-challenged group, which showed significant differences (*p* < 0.05), as determined by performing a Mantel–Cox log-rank test.

The times to the first death in the unvaccinated groups challenged with MDV alone and co-challenged with MDV and REV was 27 dpc and 17 dpc, respectively. The survival time of diseased chickens in the MDV and REV co-challenged group was generally shorter than that of diseased chickens in the MDV-challenged control group, as shown by the survival curves (Figure 1). Furthermore, the mortality patterns in the MDV-challenged control group and co-challenged group showed significant differences (*p* < 0.05).

### 3.5. Distribution of Visible Tumors

The presence of tumors in the visceral organs of experimental chickens as determined in autopsy examinations is presented in Table 2. MDV infection induced tumor development in 53.3% of unvaccinated SPF chickens, among which 23.3% had visible tumors in >1 visceral organ. These tumors were most commonly found in the liver (36.7%) and proventriculus (30.0%), followed by the spleen (16.7%), heart (6.7%), and kidney (6.7%). In contrast, the tumor rate of MDV and REV co-challenged unvaccinated chickens (80.0%) was much higher than that of chickens challenged with MDV alone, and the prevalence of multiple tumors increased by 33.3% to 56.6%. The unvaccinated, MDV and REV co-challenged chickens exhibited tumors commonly in the liver (73.3%) and kidneys (60.0%), followed by the proventriculus (56.6%), spleen (43.3%), and gonads (6.7%). These results indicated that MDV and REV co-infection significantly increased the rate of tumor formation and changed the distribution of visible tumors in SPF chickens, compared to those infected with MDV alone. No visible tumorigenesis was observed in the REV-challenged control group or in the negative control group during the experimental period.

### 3.6. Histological Lesions in Collected Tissues

**Figure 2:**
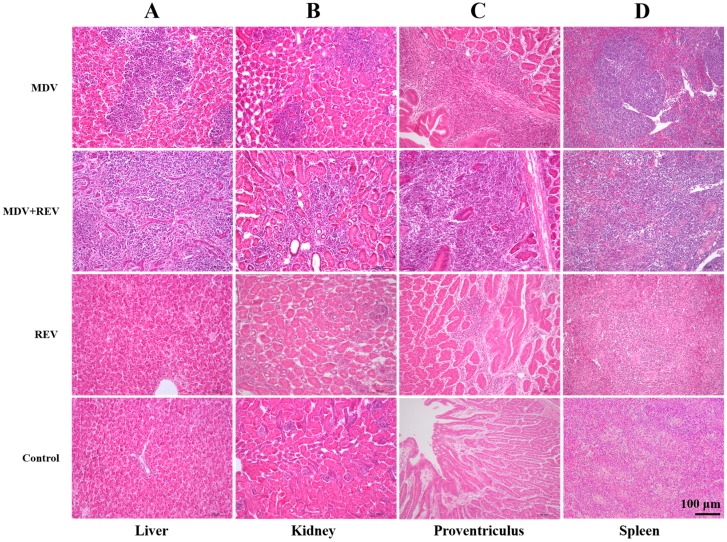
Histological lesions with hematoxylin and eosin staining of collected tissues at 200× magnification. (**A**) Liver specimens from two chickens that died at 63 dpc in the MDV-challenged control group and the MDV and REV co-challenged group, and from two chickens sacrificed humanely at the end of the animal experiments in the REV-challenged control group and the negative control group, as indicated. (**B**) Kidney specimens from two chickens that died at 73 dpc in the MDV-challenged control group and the MDV and REV co-challenged group, and from two chickens sacrificed humanely at the end of the animal experiments in the REV-challenged control group and the negative control group, as indicated. (**C**) Proventriculus specimens from two chickens that died at 65 dpc in the MDV-challenged control group and the MDV and REV co-challenged group, and from two chickens sacrificed humanely at the end of the animal experiments in the REV-challenged control group and the negative control group, as indicated. (**D**) Spleen specimens from two chickens that died at 55 dpc in the MDV-challenged control group and the MDV and REV co-challenged group, and from two chickens sacrificed humanely at the end of the animal experiments in the REV-challenged control group and the negative control group, as indicated. Scale bar: 100 µm.

During the entire experimental period, the tumor tissues from MDV-challenged chickens and chickens co-challenged with MDV and REV were collected and examined histologically. The histological results of four internal organs with a high incidence of tumors are shown in Figure 2. As shown, many differently sized tumor tissue proliferations existed in the liver parenchyma of the selected dead chickens from the MDV and REV co-challenged group and the MDV-challenged control group (Figure 2A). Numerous proliferating tumor cells were concentrated in the kidney tissues of the selected dead chickens in the MDV and REV co-challenged group and the MDV-challenged control group (Figure 2B). The infiltration of many diffuse lymphocytomas was found in the mucous membrane layer of the proventriculus of the selected dead chickens from the MDV and REV co-challenged group and the MDV-challenged control group (Figure 2C). The boundary between red pulp and white pulp and the inherent structures in the spleen tissues disappeared, and diffuse infiltration of lymphocytoma in the spleen tissues of the selected dead chickens from the MDV and REV co-challenged group and the MDV-challenged control group was apparent (Figure 2D). Furthermore, the invasion and proliferation of tumor cells in the tissues of MDV and REV co-challenged chickens were generally more severe than those in the tissues of MDV-challenged chickens. No histological lesions were observed with chickens in the negative control group, and slight lymphocytic infiltrates were observed in the proventriculus and spleen of chickens in the REV-challenged control group.

### 3.7. Efficacy of MD Vaccines

After vaccination with the MD vaccines CVI988 or 814, the morbidity rates in chickens challenged with MDV decreased by 80.0% and 90.0% to 20.0% and 10.0%, respectively, whereas these same morbidity rates in the MDV and REV co-challenged chickens decreased by 46.7% and 76.7% to 53.3% and 23.3%, respectively. Thus, the vaccine PIs of CVI988 and 814 against MDV ZW/15 were 80.0 and 90.0, respectively. However, in SPF chickens co-challenged with MDV and REV, the efficacy of both vaccines against MDV dropped dramatically: CVI988 (PI = 46.7); 814 (PI = 76.7) (Table 1).

Compared to the MDV-challenged unvaccinated group, mortality in MDV-challenged chickens vaccinated with CVI988 or 814 decreased to 16.7 and 6.7% (Table 1), respectively, and the vaccination significantly prolonged the survival time of MDV-infected chickens (*p* < 0.05, Figure 1). Co-infection of CVI988- or 814-vaccinated chickens with MDV and REV reversed that trend, increasing the mortality in both groups by 13.3 to 30.0% and 20.0% (Table 1), respectively, and the survival time of diseased chickens was shortened significantly compared to that of the corresponding vaccinated, MDV-challenged group (*p* < 0.05, Figure 1). Taken together, these findings demonstrated that co-infection with REV significantly reduced the efficacy of MD vaccines in SPF chickens.

### 3.8. MDV Genome Load in Chicken Feather Pulps and the Spleen

**Figure 3:**
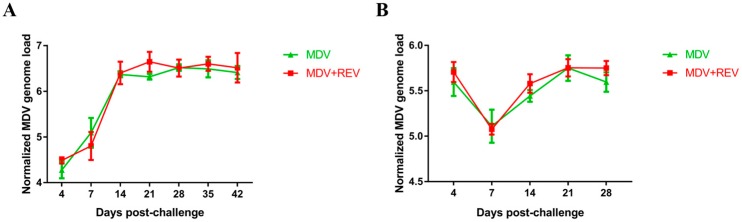
MDV genome loads of five birds from the MDV-challenged control group and the MDV and REV co-challenged group at different time points. The MDV genome loads were calculated as the logarithm of the MDV copy numbers per million cells and are shown as mean ± standard deviation. (**A**) Normalized viral loads in feather pulps; (**B**) Normalized viral loads in spleen.

To analyze the effects of REV infection on the replication of MDV in vivo, the MDV genome load in chicken feather pulps and spleen in the MDV-challenged control group and the MDV and REV co-challenged group were detected over time by qPCR (Figure 3). The viral load in chicken feather pulps of MDV in the MDV-challenged control group rose between 4 and 14 dpc, and then tended to remain stable until 42 dpc (Figure 3A). Interestingly, the viral load of MDV in chicken feather pulps in the MDV and REV co-challenged group showed a similar trend, and no significant differences (*p* > 0.05, for each comparison) were observed between the two groups at any time point. Similarly, no differences (*p* > 0.05, for each comparison) were observed in the MDV genome load in the spleen between the two groups (Figure 3B). These results suggested that REV infection has no discernable effect on the replication and proliferation of MDV in chicken feather pulps or in the spleen.

### 3.9. Differential Expression of MDV Genes in the Spleen

**Figure 4:**
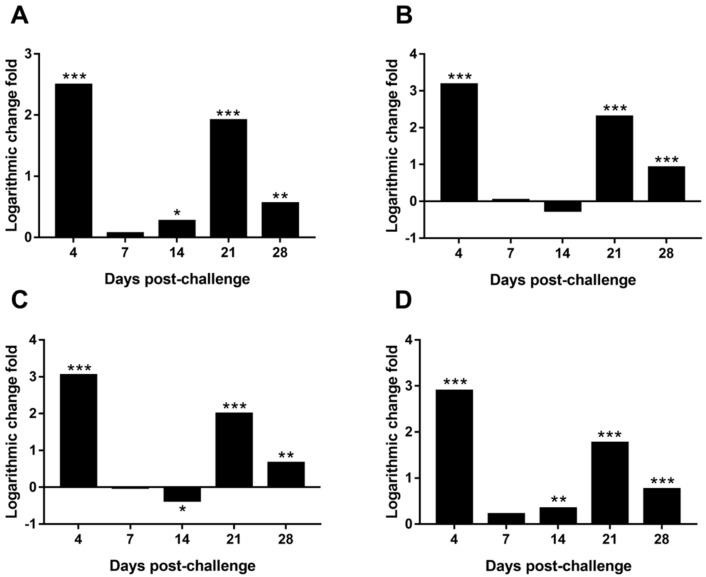
Differential expression of MDV genes in the spleens of the MDV and REV co-challenged group compared to the MDV-challenged control group. The fold-change between both groups is shown using a logarithmic scale. (**A**) Differential expression of the *meq* gene; (**B**) Differential expression of the *pp38* gene; (**C**) Differential expression of the *vIL-8* gene; (**D**) Differential expression of the *ICP4* gene. * *p* < 0.05; ** *p* < 0.01; *** *p* < 0.001.

As shown in Figure 4, 4 genes (*meq*, *pp38*, *vIL-8*, and *ICP4*) associated with MDV pathogenicity were expressed differentially in the spleen between the MDV and REV co-challenged group and the MDV-challenged control group. Specifically, expression of the *meq* gene was significantly upregulated at 4 (*p* < 0.001), 14 (*p* < 0.05), 21 (*p* < 0.001), and 28 dpc (*p* < 0.01) in the MDV and REV co-challenged group compared to the MDV-challenged control group (Figure 4A). Expression of the *pp38* gene was significantly upregulated at 4 (*p* < 0.001), 21 (*p* < 0.001), and 28 dpc (*p* < 0.001 of each comparison) in the MDV and REV co-challenged group compared to the MDV-challenged control group (Figure 4B). Expression of the *vIL-8* gene was significantly upregulated at 4 (*p* < 0.001), 21 (*p* < 0.001), and 28 dpc (*p* < 0.01) and significantly downregulated at 14 dpc (*p* < 0.05) in the MDV and REV co-challenged group compared to the MDV-challenged control group (Figure 4C). Expression of the *ICP4* gene was significantly upregulated at 4 (*p* < 0.001), 14 (*p* < 0.01), 21 (*p* < 0.001), and 28 dpc (*p* < 0.001) in the MDV and REV co-challenged group, compared with that in the MDV-challenged control group (Figure 4D). These results suggest that MDV and REV co-infection in SPF chickens significantly promoted the expression of the pathogenicity-associated MDV genes at the mRNA level at some time points during the progression of viral infection.

## 4. Discussion

The causes of oncosis in Chinese chicken flocks are complicated [51,63,64]. MD is a severe neoplastic disease caused by MDV that adversely affects the poultry industry in China [21,24,25]. Chinese chicken flocks are commonly found to be co-infected with MDV and the retrovirus REV [54,55,56,57]. However, little information is available regarding the co-infection of Chinese field MDV and REV strains, resulting in a limited understanding of the potential threat that co-infection with both viruses poses to the poultry industry. Thus, a full comprehension of the synergistic pathogenic effects between these pathogens is necessary. In this study, the pathogenicity of co-infection by Chinese field strains of MDV (ZW/15) and REV (JLR1501) in SPF chickens was compared to the pathogenicity caused by infection with MDV or REV alone.

Previously, it was found that host immune responses to REV infection were not affected by the MD vaccine [40]. In this study, our results showed that the REV-positive rate at the end of the experiment was unaffected by the status of MDV infection or MD vaccination. Therefore, we hypothesized that the effect of co-infection on RE development may be weak. Significantly, we found that co-infection with MDV and REV shortened the survival time of diseased chickens (*p* < 0.05; Figure 1) and ultimately increased the mortality (*p* < 0.05; Table 1) compared to MDV infection alone. Tumor formation was more serious in chickens of the co-infected group compared to the MDV-infection group (*p* < 0.05; Table 2 and Figure 2). These results showed that co-infection with Chinese field strains of MDV and REV could significantly aggravate the severity of illness.

The inhibitory effects on the vaccinal immunity of the HVT MD vaccine by REV infection are well elucidated [40,41]. However, at present, in China, the poultry enterprises usually implement vaccination on laying and breeding chickens using the CVI988 or 814 vaccines, and the HVT vaccine is only used to vaccinate meat-producing chickens, which have a low risk for MD. Thus, knowledge of the depressive ability of REV infection on the protective efficacy of existing MD vaccines need to be extended. In this study, we evaluated the protective efficacy of MD vaccines CVI988 and 814 against MDV under the conditions of single MDV infection or MDV and REV co-infection. Our results showed that co-infection with MDV and REV significantly reduced the protective efficacy of both MD vaccines, compared to chickens infected with MDV alone (Table 1).

Although our study covered a very specific case of co-infection with MDV and REV (simultaneous co-infection at high doses of both viruses), our findings clearly demonstrated that co-infection of MDV and REV poses a potentially serious threat to the poultry industry, as such co-infection could significantly increase the disease severity of infected chickens and reduce the vaccine protection against MD. However, little is still known about the mechanisms underlying the synergistic pathogenicity of MDV and REV. In this study, we performed the first comparison between the MDV load in chickens in a group infected with MDV alone and a MDV and REV co-infection group. Our results revealed no difference in the MDV genome load in the feather pulps and spleen between the MDV and REV co-infected chickens and the MDV-infected chickens (*p* > 0.05; Figure 3). We further examined the expression of four MDV pathogenic genes (*meq*, *pp38*, *vIL-8*, and *ICP4*) in the spleen among the MDV and REV co-infected chickens and MDV-infected chickens. The results showed that all four genes were upregulated significantly at 4, 21, and 28 dpc in the MDV and REV co-challenged group, compared to the MDV-challenged group (*p* < 0.05; Figure 4). Our findings indicated that MDV and REV co-infection might increase disease severity by increasing the expression of pathogenic genes, rather than by enhancing viral replication. Although these findings contribute to our understanding of the synergistic pathogenic mechanisms of MDV and REV, the detailed mechanism by which that co-infection promotes pathogenic MDV gene expression remains unclear. We hypothesize that REV-encoded proteins indirectly affect the expression of MDV genes by interacting with certain host cell pathways; further research on this topic is required in the future.

Our research demonstrates the effectiveness of a candidate model for studying the outcome of co-infection with MDV and REV in SPF chickens. Using this infection model, we elucidated synergistic pathogenic effects existing between MDV and REV, as well as inhibitory effects on the protective efficacy of existing MD vaccines caused by REV infection. In addition, we found that concurrent infection of REV and MDV did not affect the replication of MDV in vivo, but did affect the expression of several MDV genes. Although we achieved some interesting results, several unresolved questions remain, such as why REV and MDV co-infected chickens showed more severe gross MD lesions and why MD vaccines could not provide effective protection against MD in REV co-infected chickens. We suggest that REV-induced immunosuppression plays a major role in these processes. Previous data have shown that REV infection results in atrophy of the thymus and bursa of Fabricius in susceptible chickens [42]. The thymus and bursa of Fabricius, as central immune organs of chickens, are crucial for the development and maturation of immune cells and even the establishment of a complete immune system in the hosts. Although we are not fully aware of the host immunity to MD, several studies have shown that factors participating in host innate immunity (i.e., interferons, macrophages, dendritic cells, and natural killer cells) and adaptive immunity (both B lymphocyte-mediated antibody responses and T lymphocyte-mediated cellular immune responses) are involved in the pathogenesis of MDV infection and MD vaccine-induced immune protection [16,17]. Thus, immunosuppression induced by REV infection combined with immunosuppression and pathogenicity induced by MDV infection itself can seriously impair the host immune resistance to MDV infection, inevitably resulting in more serious illness in the co-infected chickens. Similarly, REV-induced immunosuppression inhibited the host immune activity, thereby reducing immune responses to stimulation with MD vaccines [40,41,42], by which the host could resist MDV infection and pathogenesis to some extent. Finally, REV infection caused a failure of MD vaccines to provide efficient protection against MD in such co-infected chickens. Of course, under these circumstances, many specific mechanisms remain unclear. The host immunity to MD and the mechanism of MD vaccine-induced protection remain the focus of future research on MDV. Further research into the complex interactions between MDV, REV, and susceptible hosts is also necessary.

Usually, REV infection in chicken flocks is mainly due to REV contamination in poultry vaccines [42,47,65,66]. Previous findings have shown that REV strains isolated from wild birds shared high sequence similarity with REV strains isolated from chickens [37,39], suggesting that the wild birds might also be a potential propagation source for REV. Thus, for the prevention and control of MD and RE, we should not only pay attention to the transmission and infection of MDV and REV between commercial poultries, but also between wild hosts that carry REV as a potential cause of RE. In addition, REV contamination in poultry vaccines must be strictly eliminated. Multi-level monitoring measures that target the pathogens, wild hosts, and vaccine quality will reduce MDV and REV co-infection, and effectively control economic losses to the poultry industry.

## 5. Conclusions

Our findings clearly elucidated synergistic pathogenic effects existing between MDV and REV, as well as the reduced protective efficacy of existing MD vaccines CVI988 and 814 in co-infected chickens. We also found a dynamic similarity of MDV replication among the chickens infected with MDV or co-infected with MDV and REV, and the up-regulated expression of several MDV pathogenicity-related genes in the MDV and REV co-infected chickens, for the first time. However, the exact mechanisms underlying these effects remain unclear and require further study.

## Figures and Tables

**Table 1:** Morbidity of Marek’s disease and mortality in each group.

Vaccine	Challenge	MD Incidence Diseased/Total (%)	PI	Mortality *Deaths/Total* (%)	Time (dpc) ^a^
-	-	0/15 (0%)	-	0/15 (0%)	-
-	REV	0/15 (0%)	-	0/15 (0%)	-
-	MDV	30/30 (100%)	-	23/30 (76.7%)	27
CVI988	MDV	6/30 (20.0%)	80.0	5/30 (16.7%)	51
814	MDV	3/30 (10.0%)	90.0	2/30 (6.7%)	72
-	MDV + REV	30/30 (100%)	-	29/30 (96.7%)	17
CVI988	MDV + REV	26/30 (53.3%)	46.7	9/30 (30.0%)	30
814	MDV + REV	7/30 (23.3%)	76.7	6/30 (20.0%)	28

^a^ Time that the first death occurred in each group. dpc: Days post-challenge; MD: Marek’s disease; MDV: Marek’s disease virus; PI: Protective index; REV: Reticuloendotheliosis virus.

**Table 2:** Anatomical distribution of visible tumors in each group.

Vaccine	Challenge	Total (%) ^a^	Multiple Tumors (%) ^b^	Proventriculus (%) ^c^	Heart (%) ^c^	Liver (%) ^c^	Spleen (%) ^c^	Kidney (%) ^c^	Gonad (%) ^c^
-	-	0	0	0	0	0	0	0	0
-	REV	0	0	0	0	0	0	0	0
-	MDV	53.3	23.3	30.0	6.7	36.7	16.7	6.7	0
CVI988	MDV	10.0	10.0	0	0	10.0	10.0	0	0
814	MDV	6.7	0	3.3	0	3.3	0	0	0
-	MDV + REV	80.0	56.6	56.6	0	73.3	43.3	60.0	6.7
CVI988	MDV + REV	6.7	6.7	6.7	0	6.7	6.7	0	0
814	MDV + REV	16.7	16.7	16.7	0	16.7	16.7	16.7	0

^a^ Total tumor rate in each group; ^b^ Percentage of chickens who developed tumors in more than one visceral organ; ^c^ Percentage of chickens who developed tumors in specific visceral organs.

## Supplementary Materials

The following are available online at www.mdpi.com/1999-4915/9/6/158/s1. Figure S1. Identification of MDV and REV infection in CEFs by performing IFAs, Figure S2. Detection of MDV and REV by PCR, Table S1: Primers used in the PCR amplifications for the detection of MDV and REV, Table S2: Probes and primers for RT-qPCR.
