# Supplementary material for: Co-Infection with Marek’s Disease Virus and Reticuloendotheliosis Virus Increases Illness Severity and Reduces Marek’s Disease Vaccine Efficacy
Source: Viruses. 2017 Jun 21;9(6):158. doi: 10.3390/v9060158 (PMC5490833; doi:10.3390/v9060158)
Supplement: Supplementary file 1 [file viruses-09-00158-s001.pdf]

Supplementary Materials: Co-Infection with Marek's Disease Virus and Reticuloendotheliosis Virus Increases Illness Severity and Reduces Marek's Disease Vaccine Efficacy

Guo-Rong Sun, Yan-Ping Zhang, Lin-Yi Zhou, Hong-Chao Lv, Feng Zhang, Kai Li, Yu-Long Gao, Xiao-Le Qi, Hong-Yu Cui, Yong-Qiang Wang, Li Gao, Qing Pan, Xiao-Mei Wang and Chang-Jun Liu

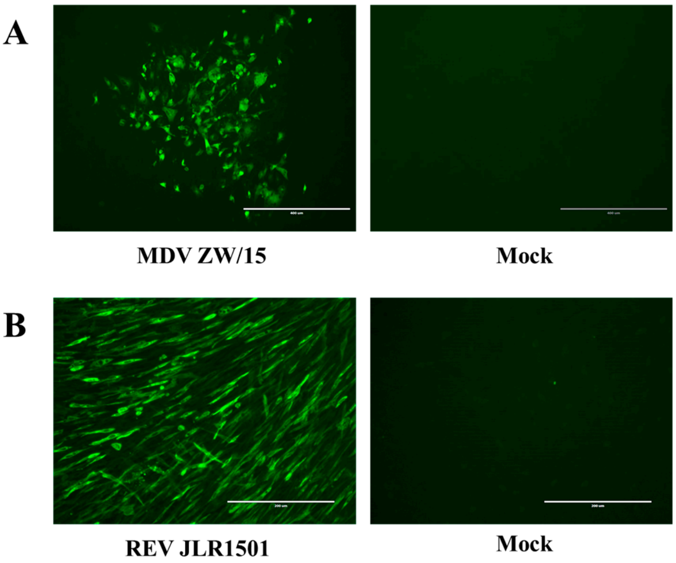

Figure S1. Identification of MDV and REV infection in CEFs by performing IFAs. (A) Viral plaques in CEFs caused by MDV ZW/15 infection were evident using an MDV gE-specific monoclonal antibody. Scale bar: 400 µm. (B) REV JLR1501 infection in CEFs was observed using an REV gp90-specific monoclonal antibody. Scale bar: 200 µm.

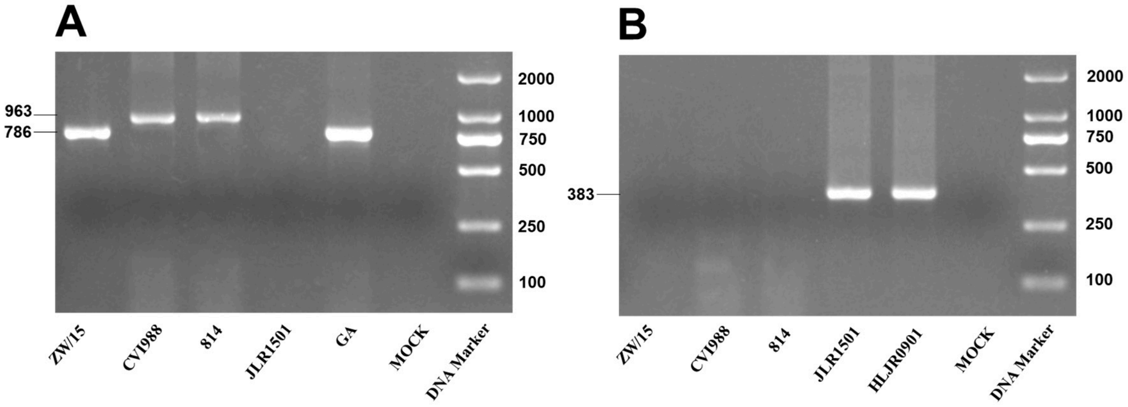

Figure S2. Detection of MDV and REV by PCR. (**A**) PCR amplifications targeting the MDV meq gene. (**B**) PCR amplifications targeting the REV ENV gene.

Table S1. Primers used in the PCR amplifications for the detection of MDV and REV.

Target	Sequence	Product (bp)
MDV meq	F: 5′-TTCCCTGACGGCCTATCTGA-3′<br>R: 5′-TTCGGGATCCTCGGTAAGAC-3′	786 or 963[a]
REV ENV	F: 5′-GCCTTAGCCGCCATTGTA-3′<br>R: 5′-CCAGCCAACACCACGAACA-3′	383

MDV: Marek's disease virus; REV: Reticuloendotheliosis virus. [a] Exact size depends on the MDV strain (some strains have a 177 bp insertion in the *meq* gene).

Table S2. Probes and primers for RT-qPCR.

Target	Probe or primer	Sequence	Accession No.
28S	Probe	5′-(FAM)-GCATGGCTTAATCTTTGAGACAA-(BHQ1)-3′	X59733
	F	5′-ATCCTGCCAGTAGCATATG-3′	
	R	5′-GCCGTGCGTACTTACACGT-3′	
meq	Probe	5′-(FAM)-CGTCTTACCGAGGATCCCAGGATCCCGAACAGG-(BHQ1)-3′	DQ453117.1
	F	5′-GGAGCCGGAGAGGCTTTATG-3′	
	R	5′-ATCTGGCCCGAATACAAGGAA-3′	
pp38	Probe	5′-(FAM)-CGGGGACGCCGAGATGAGC-(BHQ1)-3′	KC511813.1
	F	5′-CGCCTTCCCATCACCCTT-3′	
	R	5′-CCGAGGACGGCGAGAAAT-3′	
vIL-8	Probe	5′-(FAM)-CCCCAAGCCAGTAGGCCGATTA-(BHQ1)-3′	HQ638182.1
	F	5′-AATAAACTGCTGTACCCAAGGC-3′	
	R	5′-CTCGCTGTCGGCAAGAGG-3′	
ICP4	Probe	5′-(FAM)-TCCCGACTCGTGCTTTCTGTGC-(BHQ1)-3′	AF147806.2
	F	5′-CTGGGTGGGCTGTAAAATCTG-3′	
	R	5′-GAGGGCGGGAGTAGGGAG-3′	

FAM: 6-Carboxyfluorescein; BHQ1: Black Hole Quencher1.
